# Hydroxyproline-*O*-Galactosyltransferases Synthesizing Type II Arabinogalactans Are Essential for Male Gametophytic Development in Arabidopsis

**DOI:** 10.3389/fpls.2022.935413

**Published:** 2022-06-14

**Authors:** Dasmeet Kaur, Diana Moreira, Sílvia Coimbra, Allan M. Showalter

**Affiliations:** ^1^Department of Environmental & Plant Biology, Ohio University, Athens, OH, United States; ^2^Molecular and Cellular Biology Program, Ohio University, Athens, OH, United States; ^3^Departamento de Biología, Faculdade de Ciências da Universidade do Porto, Porto, Portugal; ^4^LAQV Requimte, Sustainable Chemistry, Universidade do Porto, Porto, Portugal

**Keywords:** arabinogalactan-proteins, hydroxyproline-galactosyltransferases, pollen grains, microgametogenesis, exine, intine, pollen tube

## Abstract

In flowering plants, male reproductive function is determined by successful development and performance of stamens, pollen grains, and pollen tubes. Despite the crucial role of highly glycosylated arabinogalactan-proteins (AGPs) in male gamete formation, pollen grain, and pollen tube cell walls, the underlying mechanisms defining these functions of AGPs have remained elusive. Eight partially redundant Hyp-galactosyltransferases (named GALT2-GALT9) genes/enzymes are known to initiate Hyp-*O*-galactosylation for Hyp-arabinogalactan (AG) production in *Arabidopsis thaliana*. To assess the contributions of these Hyp-AGs to male reproductive function, we used a *galt2galt5galt7galt8galt9* quintuple *Hyp-GALT* mutant for this study. Both anther size and pollen viability were compromised in the quintuple mutants. Defects in male gametogenesis were observed in later stages of maturing microspores after meiosis, accompanied by membrane blebbing and numerous lytic vacuoles. Cytological and ultramicroscopic observations revealed that pollen exine reticulate architecture and intine layer development were affected such that non-viable collapsed mature pollen grains were produced, which were devoid of cell content and nuclei, with virtually no intine. AGP immunolabeling demonstrated alterations in cell wall architecture of the anther, pollen grains, and pollen tube. Specifically, the LM2 monoclonal antibody (which recognized β-GlcA epitopes on AGPs) showed a weak signal for the endothecium, microspores, and pollen tube apex. Pollen tube tips also displayed excessive callose deposition. Interestingly, expression patterns of pollen-specific AGPs, namely AGP6, AGP11, AGP23, and AGP40, were determined to be higher in the quintuple mutants. Taken together, our data illustrate the importance of type-II AGs in male reproductive function for successful fertilization.

## Introduction

The evolutionary success of angiosperms is usually ascribed to their complex double fertilization process and the coordinated activity of the developing gametophytic tissues within the diploid sporophytic reproductive organs. Both the male (microspores) and female (embryo sac) gametophytes develop during two similar phases: microsporogenesis and microgametogenesis for pollen development in the sporophytic diploid tissue of stamen, megasporogenesis, and megagametogenesis for the embryo sac development inside the diploid maternal organ called the ovule. Such closely controlled gametophytic development in flowering plants involving proper pattern formation, cell speciation, cell count, and cell polarity ensures reproductive success (Berger et al., 2008; Borg et al., 2009; Sprunck and Groß-Hardt, 2011; Schmid et al., 2015; Hafidh et al., 2016; Higashiyama and Yang, 2017).

Unlike animals, plants have highly reduced immotile sperm cells that exist within a pollen grain and a pollen tube cell, which grows and releases the two sperm cells into the embryo sac for double fertilization. Thus, highly orchestrated male gametophytic development is comprised of two consecutive phases, namely the developmental phase and the functional/progamic phase. The developmental phase of male gametes proceeds inside anther loculi surrounded by four somatic cell layers, namely the tapetum, middle layer, endothecium, and epidermis (Goldberg et al., 1993; Sanders et al., 1999). Pollen development and pollen cell wall development were recently reviewed in detail by Ma et al. (2021). During this process, the microsporocytes encapsulated inside the anther loculi undergo meiosis forming tetrads with high callose deposition between each of these four microspores. Meanwhile, the secretory tapetal cells that differentiate into metabolically active binuclear cells become rich in proteins, lipids, carbohydrates, and secondary metabolites and nourish these developing microspores (Hsieh and Huang, 2005; Pacini et al., 2006; Li et al., 2012). Tight regulation of tapetal cell development and their programmed cell death is coordinated with other microspore developmental processes (Mariani et al., 1990; Ariizumi and Toriyama, 2011; Lou et al., 2014; Li D.D. et al., 2017). Microspores undergo remarkable morphological and physiological differentiation, including biosynthesis of a unique pollen cell wall. Highly ordered pollen wall formation is initiated at the tetrad stage where a dense fibrous structure known as the primexine (consisting of protein, callose, and acidic polysaccharides), is laid down on the microspore surface. The primexine provides the framework for the arrangement of the tectum and bacula. Later, the protein and lipid-rich pollen coat obtained from enzymatic catalysis of the tapetum is deposited over the exine cavities of the outer exine layer (Ariizumi and Steber, 2007; Liu and Fan, 2013; Quilichini et al., 2015), followed by the release of mature pollen grains with a vegetative cell and two sperm cells. The multilayered pollen cell wall performing the highly specialized biological role in fertilization is composed of the outer exine layer (mostly of sporophytic origin) and the inner pectocellulosic intine layer (of gametophytic origin; Wang et al., 2018; Grienenberger and Quilichini, 2021). Further, the exine consists of two layers, the inner nexine and outer sexine. The three-dimensional homogenous latticework of sexine reticulate architecture is precisely laid out by its elements: baculae rising like columns and tecta forming the roofs on these columns (Suzuki et al., 2017). The nexine is composed of a sporopollenin-rich outer nexine I (foot of probacula), and an inner nexine II (Ariizumi and Toriyama, 2011; Ma et al., 2021).

The functional/progamic phase is subsequently initiated with the landing of the sticky pollen grain on the stigmatic surface. Through rehydration, the pollen grain gets activated and germinates into a pollen tube penetrating the pistil tissues (Cheung et al., 1995; Wu et al., 1995). Pollen tube guidance of compatible pollen grains, the communication between the two gametophytes and pollen tube reception are key advancements that occur prior to the fusion of the sperm cells into the egg cell and central cell. In addition to playing a critical role in sexual reproduction, pollen grains also provide an attractive model system to study the role of localized interactive molecules like arabinogalactan-proteins (AGPs) in the developmental regulation of cell morphogenesis and differentiation of microspores.

AGPs represent a family of structurally complex, highly glycosylated hydroxyproline-rich proteins that are found at the plasma membrane-cell wall interface, in the cell wall, and in plant exudates of virtually all plant cells. The protein backbones of AGPs are characterized by an abundance of proline/hydroxyproline (Hyp), serine, alanine, and threonine residues. Being extensively modified by type II arabinogalactan polysaccharides (type-II AGs; ∼90–98% of w/w) on non-contiguous Hyp residues, make AGPs biochemically information-rich molecules that presumably are involved with interactions with other cell surface components for cellular signaling. Multiple AGPs are highly expressed during male gametophytic development, pollen cell wall, and pollen tube growth. These AGPs are implicated in the formation of healthy pollen grains, enhancing germination percentages (Coimbra and Gustavo, 2012), and likely the pollen tube cell capacity to perceive stylar and ovular signaling cues for double fertilization (Cheung et al., 1995; Pereira et al., 2016). For instance, AGP6 and AGP11 are implicated in gametophytic pollen biogenesis (Coimbra et al., 2009). In addition to AGP6 and AGP11, the expression of AGP23 and AGP40 is regulated to form the nexine layer during pollen grain development (Pereira et al., 2014; Jia et al., 2015). Mutants for AtFLA3 showed defects in the pollen intine layer and in pollen germination (Li et al., 2010), while AtFLA14 mutants showed collapsed pollen grains and precocious pollen germination under high moisture conditions (Miao et al., 2021). Notably, a mutation in the *KNS4/UPEX1* gene which encodes β-(1,3)-galactosyltransferase activity (GT31 family) for AGPs, resulted in abnormal primexine development (Suzuki et al., 2017). Furthermore, CRISPR/Cas9 mutants in the *GLCAT14A-C* genes (GT14 family), which encode GlcA transferase activity for AGPs, produced plants having reduced yields due to the intine and exine defects in the mature pollen grains (Zhang et al., 2020; Ajayi et al., 2021).

Out of the 25 known glycosyltransferases (GTs) involved in the synthesis of type-II AGs, eight hydroxyproline-*O*-galactosyltransferases (i.e., GALT2-9), belonging to the CAZy GT31 family, have been characterized to add the first galactose sugars to AGP protein backbones and initiate AGP glycosylation (Basu et al., 2015a,b; Ogawa-Ohnishi and Matsubayashi, 2015). Being members of a multigene family, partial-redundancy thwarted efforts to obtain any discernable reproductive phenotypes in single or double mutants of the *GALT2-9* genes. To overcome this issue, we generated a quintuple *galt25789* mutants that showed a substantial effect on the reproductive ability along with the observation of aborted pollen exhibiting exine structural differences (Kaur et al., 2021). Additionally, *galt23456* CRISPR/Cas9 mutants also revealed aborted pollen grains to be responsible for reduced seed set in a previous study (Zhang et al., 2021). Here we used various microscopic and immunological techniques to provide deeper insight into the male gametophytic defects in the *galt25789* mutant. In this study, we investigated the role of type-II AGs of AGPs in male sporophytic and gametophytic development, pollen grains, and pollen tube growth by using the *galt25789* mutant.

## Materials and Methods

### Source and Plant Growth Conditions

*Arabidopsis thaliana* (Columbia-0 ecotype) was used as the WT and was obtained from the Arabidopsis Biological Research Center (ABRC), Columbus, OH, United States. The *galt2 galt5 galt7 galt8 galt9* (*galt25789*) mutants were generated from T-DNA insertional mutants using a traditional crossing strategy described previously (Kaur et al., 2021). For this study, the *galt2 galt5* and *galt7 galt8 galt9 (hpgt1 hpgt2 hpgt3)* mutants were used as controls. All plants (WT, *25*, *789*, and *galt25789*) used in this study were germinated after 3 days of stratification in the dark at 4°C on Murashige and Skoog medium (Caisson Laboratories, North Logan, UT, United States) containing 1% sucrose and 4 g/L Phytagel. On 7 day after germination, all plants were transplanted onto the soil and grown under long-day conditions (16 h of light/8 h of dark, 22°C, 60% humidity) in growth chambers.

### Alexander Staining

To examine whether pollen grains from *galt25789* mutant plants were viable, Alexander staining was performed as previously described by Peterson et al. (2010). The Alexander stain was prepared by mixing 10 ml of 95% ethanol, 1 ml of Malachite green (1% solution in 95% ethanol), 50 ml of deionized water, 25 ml of glycerol, 5 ml of acid fuchsin (1% solution in water), 0.5 ml of Orange G (1% solution in water), and 4 ml of glacial acetic acid in a total volume of 100 ml. Samples were stained with Alexander stain and heated to just below boiling for 30 s, rinsed, and observed with a Nikon Phot-lab2 light microscope.

### 4′,6-Diamidino-2-Phenylindole Staining

To observe the nuclei and callose wall, mature pollen grains were stained in 4′,6-diamidino-2-phenylindole (DAPI) solution (Regan and Moffatt, 1990). Briefly, the DAPI staining solution was made fresh on the day of use by adding 1.5 μl of 1 mg/ml DAPI stock solution (stored in dark) to 1 mL of sterile distilled water. Images were captured with a Nikon E600 epifluorescence microscope.

### Auramine O Staining

For auramine O staining, pollen grains of stage 13 flowers were suspended in 0.1% auramine O in 50 mM Tris–HCl, pH 7.5 and observed with a Zeiss LSM-510 laser-scanning confocal microscope at Ohio University using the filter set suitable for FITC.

### Electron Microscopy

For SEM observations, pollen grains and anthers of WT, *25*, *789* and *galt25789* were dry-mounted on aluminum stubs using double-adhesive tapes and sputter-coated with a palladium alloy using an Anatech HUMMER 6.2 Sputtering System). Images were captured using an SEM JEOL JSM-6390, HV/LV Tungsten/LaB6, Jeol USA Inc. (Hitachi High-Technologies, Japan), with an accelerating voltage of 15 kV at the Institute for Corrosion and Multiphase Technology, Ohio University. ImageJ software was used to measure the pollen area of more than 200 pollen. For TEM observations, ultrathin sections of resin-embedded anthers were prepared using a Leica EM UC6 ultramicrotome (Wetzlar, Germany) with a diamond knife and mounted on copper grids essentially as described by Suzuki et al. (2008). Specimens were viewed with a Hitachi H-7500 Transmission Electron Microscope equipped with an SIA-L12C digital camera and software at the Molecular and Cellular Imaging Center (MCIC), Ohio State University, Ohio Agricultural Research and Development Center (OARDC) in Wooster, OH.

### Immunolabeling of Anther Sections

Flower bud clusters at inflorescence apices were fixed as previously described by Suzuki et al. (2017). Briefly, fixation was performed in buffer [2% (v/v) formaldehyde, 2.5%(v/v) glutaraldehyde, 25mM Na-P buffer, pH7.5] for 24 h at 4°C, then dehydrated through an ethanol series. The ethanol was replaced with a 1:1 mix of LR White resin (type medium; Electron Microscopy Sciences) and ethanol, then with pure resin. Sections (1 μm) were cut in a microtome and mounted on MAS-coated glass slides. Sections were treated with a solution (1% (w/v) bovine serum albumin (BSA) in PBST (5.1 mM Na_2_HPO_4_, 1.6mM KH_2_PO_4_, 130mM NaCl, 0.02% Tween 20) for 1 h at RT for blocking, and subsequently incubated with a 1:10 dilution of primary antibody (JIM5, JIM7, JIM13 and JIM8 from CarboSource Services, LM2 from Plant Probes) in the same buffer. PBST buffer washing was then conducted three times. Alexa Fluor 488 Goat anti-rat fluorescein isothiocyanate (FITC)-conjugated secondary antibody (Invitrogen; diluted 1:100 in PBS in 1% BSA) was used for a 2 h incubation in the dark at RT. After washing with PBST, slides were mounted with aqua-poly/mount (Polysciences). A Nikon Eclipse E600 epifluorescence microscope was used for observations. Fluorescence of Alexa Fluor 488 and background autofluorescence of the samples were captured with FITC bandpass filter (excitation wavelength of 460–500 nm, emission wavelength of 510–560 nm) and a DAPI bandpass filter (excitation wavelength of 330–380 nm, emission wavelength of 435–485 nm), respectively. Both images were captured simultaneously to make an overlapping image with Photoshop software.

### Toluidine Blue Staining

Thick sections (1 μm) of resin-embedded anthers were mounted on a glass slide, stained with a toluidine blue staining solution [0.2% (w/v) toluidine blue, 0.5% (w/v) sodium borate], and the slides heated on a hot plate for 10 min. After washing out the stain, the specimen was viewed with a Nikon Phot-lab2 light microscope.

### Aniline Blue Staining and Immunolabeling of Pollen Tubes

Flowers collected from WT and *galt25789 Hyp-GALT* mutant plants 1–2 weeks after bolting were used for examination of pollen tube phenotypes. Individual open flowers were germinated *in vitro* as described previously (Kaur et al., 2021) with minor modifications. Briefly, a liquid germination medium contained 0.01% H_3_BO_3_, 1 mM Ca(NO_3_)_2_, 1 mM KCl, 1 mM CaCl_2_, 10% sucrose, 0.03% casein enzymatic hydrolysate, 0.01% myo-inositol, 0.1 mM spermidine, 10 mM GABA, and 500 μM methyl jasmonate, pH 7.5), and pollen tubes were grown at 22°C and 100% humidity in the dark for 6 h for both immunolabeling and aniline blue staining. Pollen tubes were fixed as described by Dumont et al. (2015). Primary antibodies were diluted at 1:5 or 1:10 as described previously with phosphate-buffered saline, PBS (with 3% milk). Pollen tubes were rinsed with the buffer and incubated overnight at 4°C in the dark with goat anti-rat IgG -FITC secondary antibody (diluted 1:50) for 3 h at 30°C. Controls were carried out by incubation of the pollen tubes with the secondary antibody only. For callose staining, pollen tubes were rinsed with PBS and stained with decolorized aniline blue (0.1%, w/v; Thermo Fisher Scientific) in 100 mM K_3_PO_4_, pH 11.

Pollen tube intensity images were analyzed using ImageJ software as described by Beuder et al. (2020). Briefly, fluorescence signal intensities were measured along both the peripheries of the pollen tubes, starting from the tip toward the pollen tube shaft. The Plot Profile tool was used to get the pixel gray value results along with the distance. The two measurements of pollen tubes were averaged out, and the measurements of all pollen tubes were plotted.

### RNA Extraction, Complementary DNA Synthesis, and Real-Time RT-PCR

Total RNA was extracted from inflorescences using PureZol RNA Isolation Reagent (Bio-Rad) following the manufacturer’s instructions. DNA was removed by DNase (Thermo Scientific) treatment. Isolated RNA samples were reverse transcribed using RevertAid First Strand cDNA Synthesis kit (Thermo Scientific) and oligo(dT)18 primers to initiate the reactions. Complementary DNA (cDNA) was amplified using the SSoFAST SYBR Green Supermix (Bio-Rad) in an iQ5 Real-Time RT-PCR (Bio-Rad) detection system using specific primers listed in Supplementary Table 1. Primers for the reference genes *ACTINA8 ACT8* (*At1g49240*) and *ARABIDOPSIS THALIANA RELATED TO UBIQUITIN 1 RUB1* (*At1g31340*) were used. Three technical replicates were performed for each situation. After 3 min at 95°C, a 10s denaturation step at 95°C was followed, and 45 cycles of 95°C at 10s and 60°C at 30s were performed. After amplification, the dissociation curve was acquired to verify the specificity of the amplification by heating the samples from 60 to 95°C. At the end of the PCR cycles, data were analyzed using the CFX Maestro™ Software (Bio-Rad) program.

## Results

### *galt25789* Mutant Has Defects in Floral Meristem Architecture, and Aberrations in Anther Morphology, Exine Patterning, and Pollen Viability

**FIGURE 1:**
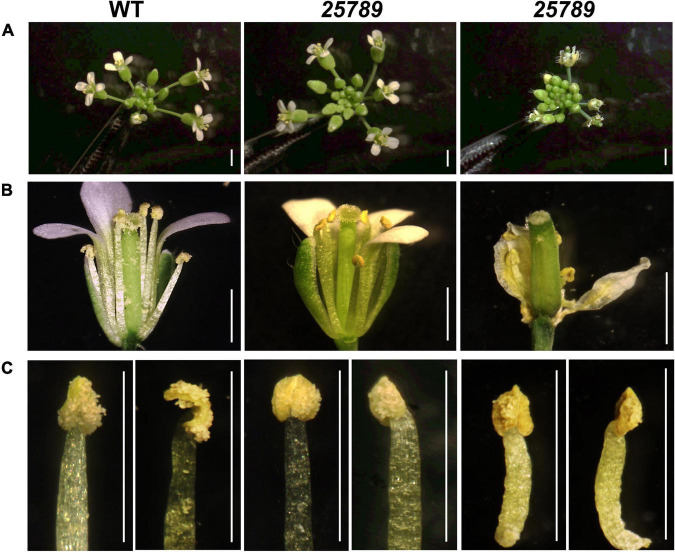
Comparison of the WT and *Hyp-GALT galt25789* quintuple mutant inflorescences and stamen. **(A)** WT and *galt25789* quintuple mutant inflorescences. **(B)** Comparison of stage 13 flowers of the WT and *galt25789* quintuple mutants. Normal inflorescences were accompanied by withered/stunted inflorescences in *galt25789* mutants. Note, the *galt25789* mutant flowers have reduced amount of pollen sticking to the style and stigma. **(C)** Anther morphology of stage 13 flowers of the WT and *galt25789* quintuple mutants with front view and side view, respectively. Scale bar = 1.0 mm.

To examine the effect of *Hyp-GALT* mutations on Arabidopsis fertilization, we started with a phenotypic examination of the inflorescences. As reported earlier, no discernible morphological changes in the flower organs of double, triple, and quadruple *Hyp-GALT* mutants compared to the WT were observed (Kaur et al., 2021); however, enlarged inflorescences with numerous buds were observed in the some of the *galt25789* mutant plants which became withered flowers or did not bloom in extreme cases (5–10%; Figure 1).

**FIGURE 2:**
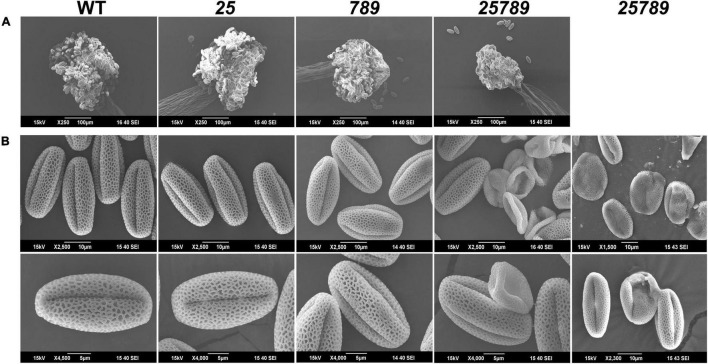
Anther and pollen grains reveal defects in mature pollen grains of *galt25789 Hyp-GALT* mutants. **(A)** SEM images of anthers from WT, *galt25* and *galt789* and *galt25789* showed that anther size is reduced in the *galt25789* mutants. **(B)** SEM images of the WT, *galt25, galt789* and *galt25789* pollen grains. *Galt25789* mutant displayed misshaped, collapsed, and defective pollen with abnormal exine patterns in comparison to regular reticulate exine structure in WT. Scale bars = 100 μm in **(A)**, 10 and 5 μm in **(B)** as indicated.

**FIGURE 3:**
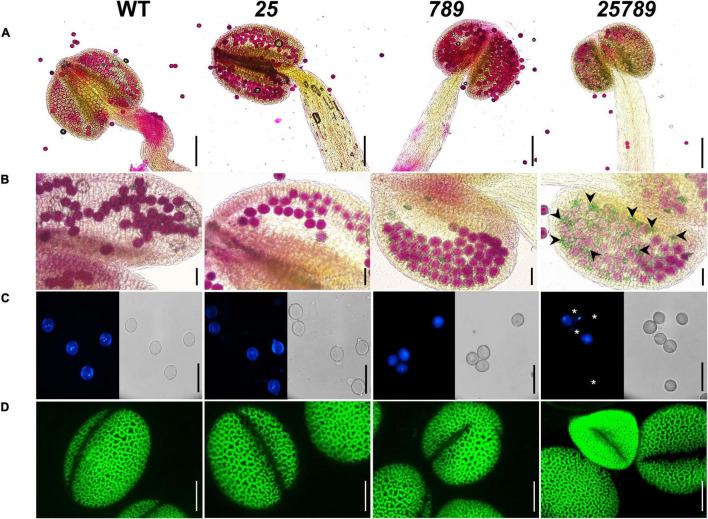
Characterization of pollen grain defects by alexander, DAPI, and auramine-O staining of *Hyp-GALT* mutants compared to WT. **(A)** Complete view of anther showing pollen viability of WT, *galt25*, *galt789*, and *galt25789*, accessed by alexander staining. (**B**) High magnification view showing alexander staining of pollen grains. *galt25789* had abnormal aborted pollen (indicated by green-colored pollen and black arrowheads in panel). **(C)** DAPI staining of mature pollen grains released from anthers, under a light microscope and a DAPI filter. Mature pollen grains detected the presence of two sperm nuclei and one vegetative nucleus in WT, *galt25* and *galt789* and *galt25789* except for the aborted pollen grains (indicated by white asterisks) in *galt25789* mutant. **(D)** Confocal images of pollen stained with 0.1% auramine-O in WT, *galt25* and *galt789* and *galt25789*. Scale bars = 200 μm in **(A)**; 50 μm in **(B,C)**; 10 μm in panel **(D)**.

Earlier, we demonstrated reduced seed-set and associated aborted pollen phenotypes in *galt25789* mutant (Kaur et al., 2021). Indeed, *in vitro* pollen germination showed a reduction of 50% relative to 77% WT germination. All the eight *Hyp*-GALTs are predicted to be expressed in various developmental stages of microspores and pollen tubes (Supplementary Figure 1). To further examine the underlying cause of male sterility associated with the *galt25789* mutant, the anther morphology of floral stage 13 *galt25789* mutant flowers were examined using both a stereomicroscope and scanning electron microscopy (SEM). In comparison to the WT, the *galt25789* mutant displayed defects in its pollen releasing capacity (Figures 1B,C) and anther size (Figures 1C, 2A and Supplementary Figure 2A). Furthermore, we observed a distorted reticulate exine pattern on *galt25789* mutant pollen grains with smaller or obstructed lacunae; the exine dissociated easily from the pollen surface making them sticky (Figure 2B). Notably, the reticulate structure of the *galt25* mutant was comparable to WT whereas *galt789* mutants were mildly affected with respect to small lacunae and some aborted pollen compared to the WT (Figure 2B). In addition, the exine-specific dye, auramine-O showed altered exine patterning in the *galt25789* mutant, specifically in collapsed pollen showcasing the ablation of reticulate design normally found in WT (Figure 3D).

We conducted Alexander staining to test pollen viability, and we observed a higher proportion of non-viable (green colored) pollen in *galt25789* mutants relative to the WT (0.5%; Figures 3A,B). About 28.2% of mutant pollen grains presented an abortion phenotype (Supplementary Figure 2B). In line with these observations, DAPI staining of pollen grains (Figure 3C) also lacked two sperm nuclei in most of the mature pollen grains with exine defects or shrunken shape. Hence, mutations in the five *Hyp-GALT* genes of *galt25789* mutant abolished the regular network of exine projections, resulting in structurally weakened pollen grains that easily collapsed/aborted.

### *Hyp-GALT* Mutations Cause Defects in Male Gametophytic Development, Pollen Exine and Intine Development Leading to a Degeneration of Developing Microspores

**FIGURE 4:**
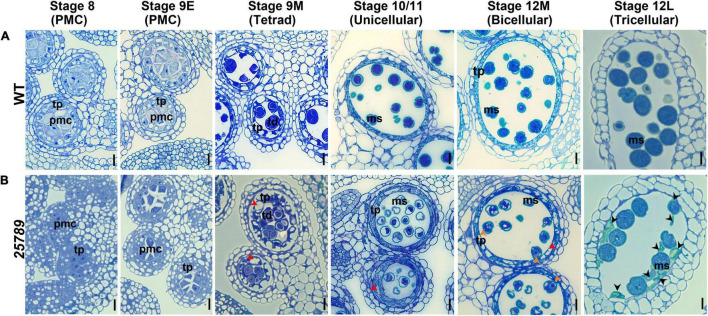
Light and TEM micrographs reveal defects in pollen development in *galt25789 Hyp-GALT* quintuple mutant. Light micrographs of cross-sections of resin-embedded anthers of WT **(A)** and *galt25789* quintuple **(B)** plants stained with toluidine blue. Developmental stages 8 to 12L of anthers along with corresponding pollen developmental stages are indicated at top. Red arrowheads in stages indicate vacuolated and thicker tapetal cell layer, as well as vacuolated pollen grains, at binucleate stage of the *galt25789* mutant. Orange arrowheads indicate cytoplasmic shrinkage of pollen grains. Black arrowheads indicate cell wall debris of crushed pollen grains. Scale bar = 10 μm. pmc-pollen mother cell; td-tetrad; tp-tapetum; ms-microspore.

To determine the underlying cytological differences and precise stages during which pollen grains aborted/collapsed, anther sections of WT and *galt25789* (Smyth et al., 1990) were examined using toluidine blue staining. WT shows normal sporophytic and gametophytic development of pollen grains (Figure 4A). The mutant microspores develop like WT by the tetrad stage and gradually started degenerating during the maturation process of microspore development. The first sign of a developmental abnormality in *galt25789* mutants appears in stage 10/11 after meiosis where the sporophytic tapetum of *galt25789* anthers appeared swollen and contained a slightly greater number of vacuoles in the tapetum layer (Figure 4B). Interestingly, *galt25789* mutant developing microspores also contained more vacuoles compared to the WT. The cytoplasmic retractions in mutant pollen grains and the vacuolated tapetal cell phenotype appeared to be more obvious in the bicellular stage when compared to WT. At the tricellular stage (12L) in WT, when tricellular mature pollen grains were developed, the tapetal cells eventually disappear leaving behind the mature pollen grains in anther locules. However, in *galt25789* mutant locules, many pollen grains adhered to each other and the endothecium surface layer, presumably due to the debris released by collapsed pollen grains that stained weakly with toluidine blue (Figure 4B).

**FIGURE 5:**
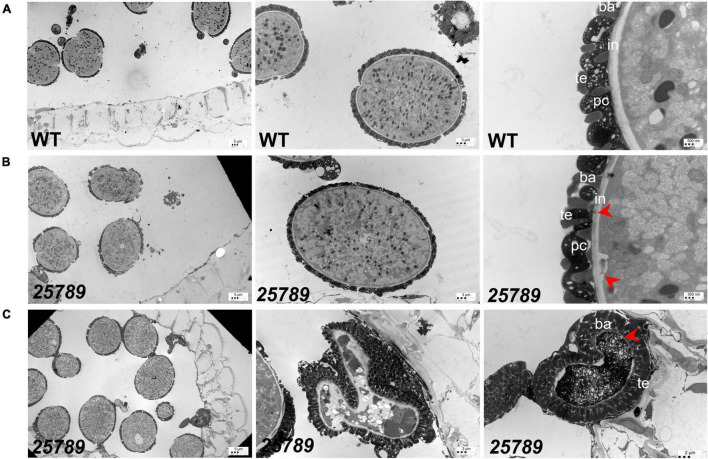
TEM images of WT and *galt25789* mutant microspores. **(A)** Microspores from WT plants. Exine, Intine, and pollen coat of pollen grains (tricellular stage) at stage 12L. **(B)** Microspores from *galt25789* mutant plants. Intine is thin and membrane blebbing is evident in *galt25789* mutant normal microspores (indicated by red arrows). **(C)** Abnormal pollen wall patterning is visible in the aborted mature pollen stage. The mature aborted pollen grain wall structure is aberrant. The pollen grain gets devoid of any content and nuclei. Intine layers are virtually absent (marked by red arrow), and a very dark cytoplasm marks the degradation of the cytoplasm. ba, baculum; ex, exine; in, intine; te, tectum; pc-pollen coat. Scale bars = 5 μm; 3 μm; 2 μm; 500 nm as indicated.

Exine structure in mature pollen grains of *galt25789* mutants was investigated by transmission electron microscopy (TEM). Aborted pollen grains presented aberrant reticulate exine patterns accompanied by extensive degeneration of all cytoplasmic contents, as they became electron dense at the binucleate stage (Figure 5C). Moreover, the intine layer of *galt25789* microspores was either unable to form or remnants of the intine layer persisted. Subsequently, degradation of the cytoplasmic content and the failure of intact intine formation likely leads to the dissolution of nuclei and microspore collapse by the dehiscent stage. On the other hand, viable *galt25789* microspores with a normal appearance contained less developed intine (Figure 5B) compared with the presence of complete intine and exine structures in WT microspores (Figure 5A).

In *galt25789*, developing microspores at the uninucleate and binucleate stages revealed many lytic vacuoles, reflecting higher metabolic activity while only vegetative vacuoles are seen in the WT (Supplementary Figure 3). The large vacuoles in which numerous membranous structures appeared to be engulfed, like autophagic bodies, were present in *galt25789*. All these results indicated that microspore abortion in *galt25789* microspores occurred at the uninucleate or binucleate stage and suggested a crucial role for Hyp-*O*-glycans in microspore development and pollen wall patterning in Arabidopsis, especially in intine formation.

### Distribution of Glycosylated Arabinogalactan-Protein and Pectin Epitopes in Developing Anthers

**FIGURE 6:**
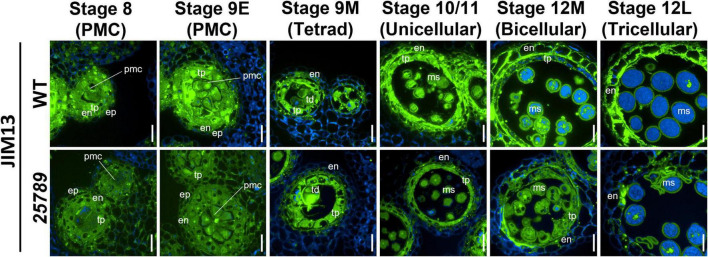
Distribution of JIM13-epitope labeling of AGPs in microsporocytes and microspores of *galt25789* mutants and WT. Cross sections of resin-embedded anthers of WT (Col-0) and *galt25789* from stages 8 to 12L were labeled with JIM13 primary antibodies followed by Alexa Fluor 488-labeled secondary antibody labeling subsequently. Fluorescence of Alexa Fluor 488 (green) and autofluorescence (blue) was separately captured by epifluorescence microscopy and merged. Scale bar = 20 μm. en-endothecium; ep-epithelium; ms-microspore; pmc-pollen mother cell; td-tetrad; tp-tapetum.

AGP localization with monoclonal antibodies (mAB) directed against AGP glycosidic epitope serve as a valuable tool to observe the distribution of AGPs in Arabidopsis anthers. To determine the effect of the *galtgalt25789* mutations during the anther development, cross-sections of young flower buds from stage 8 to stage 12M were labeled with the JIM13 (Figure 6) and J1M8 (Supplementary Figure 4) mABs that recognize AG epitopes on AGPs (Pennell et al., 1991; Yan et al., 2015). JIM13 likely identifies AGPs with β-Glc*p*A-(1→3)-α-Gal*p*A-(1→2)-L-Rha epitopes (Knox et al., 1991). At stages 8 and 9E, flower development occurs when microspore mother cells differentiate, JIM13 labeling was stronger within the cell walls, PMC cytoplasm, tapetal cells, middle layer cells, and endothecium cells of the WT compared to the *galt25789* mutant (where the JIM13 signal was detected but comparatively weaker; Figure 6) which likely reflects the presence of AGP epitopes in lower proportion in these mutants. Consistently, at stage 9M, the primary walls of the WT tetrads and the tapetal walls and at stage 10/11 (unicellular) tapetal cells and unicellular microspores exhibited bright punctate signals for JIM13 compared to the *galt25789* mutant. At stages 12M and 12L, the JIM13 signal intensity became stronger in endothecium and walls of developing microspores at the bi- and tricellular stages of the WT. Clearly, a weaker signal was observed in endothecium of the *galt25789* mutant (Figure 6). At stage 12L, the signal in viable microspore walls of *galt25789* mutant was indistinguishable from WT. Interestingly, the cell wall labeling pattern of the aborted pollen in the mutant anthers presented as more intense (see collapsed microspores in Figure 6).

Immunolabeling with the other AGP antibody, JIM8 presented a distinct labeling pattern for mature microspore walls. While the tapetal cell layers of *galt25789* mutant anthers remained indifferent to the WT tapetal layer, slightly weaker JIM8 signals were detected for bicellular microspore cell walls when compared to WT (Supplementary Figure 4). Intriguingly, in line with our previous observations with JIM13, JIM8 anti-AGP antibody specifically labeled collapsed and degraded pollen grain walls (at stage 12L) of *galt25789* mutant compared to the normal pollen grain walls (Supplementary Figure 4).

**FIGURE 7:**
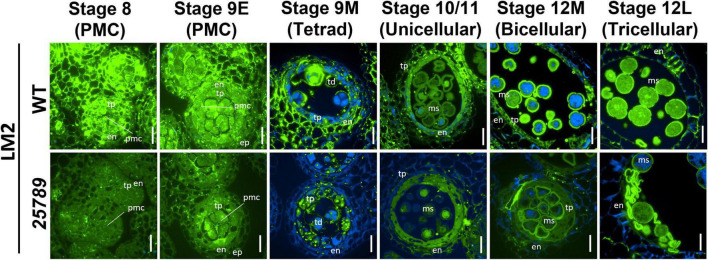
Distribution of LM2-epitope labeling of AGPs in microsporocytes and microspores of *galt25789* mutants and WT. Cross sections of resin-embedded anthers of WT (Col-0) and *galt25789* from stages 8 to 12L were labeled with LM2 primary antibodies followed by Alexa Fluor 488-labeled secondary antibody labeling subsequently. Fluorescence of Alexa Fluor 488 (green) and autofluorescence (blue) was separately captured by epifluorescence microscopy and merged. Scale bar = 20 μm. en-endothecium; ep-epithelium; ms-microspore; pmc-pollen mother cell; td-tetrad; tp-tapetum.

The use of another AGP-specific mAB, LM2, that recognizes a carbohydrate epitope containing β-D-Glc*p*A (Smallwood et al., 1996), revealed a dramatic decrease in immunolabeling intensity at all the anther developmental stages (8–12L) of *galt25789* mutant compared to the WT (Figure 7). In these developmental stages, LM2 labeling weakened in the primary cell walls in the PMC stage and the tapetal cells. In contrast to JIM13, LM2 did not present a strong signal in WT endothecium, however, the endothecium labeling slightly diminished at the tricellular stage too. A weaker exine wall signal in the viable tricellular pollen grains of *galt25789* mutant was noticed at stage 12L in comparison to WT. Interestingly, the aborted pollen grains of the mutant was more intensely labeled than the viable pollen grain walls of the WT (Figure 7).

Given the critical role of AGPs in laying the framework of the microspore cell wall during pollen development, we examined the effect that the *galt25789* mutant and its resulting AGPs had on other cell wall components, such as pectin. To examine the distribution of pectic polysaccharides, immunolabeling with two pectin monoclonal antibodies (Knox et al., 1990), JIM5 (which labels partially methylesterified pectin) and JIM7 (which labels heavily methylesterified pectin) were used. In both WT and *galt25789*, JIM7 strongly labeled the walls separating PMCs and tapetal cells at stages 8 and 9E and the primary walls of tetrads (stage 9M; Supplementary Figure 5). All anther developmental stages exhibited quite similar labeling patterns for *galt25789* mutants when compared to the WT except that the aborted *galt25789* pollen grains presented a stronger signal (Supplementary Figure 5). In contrast, the distribution of JIM5 labeling was different from that of JIM7 in WT, showing a weaker signal in stages 8, 9E, 9M, and 10/11, while stage 12M to 12L showed intense staining for the mature WT pollen grains (Supplementary Figure 6).

### Altered ImmunoLabeling and Callose Labeling of Pollen Tubes

**FIGURE 8:**
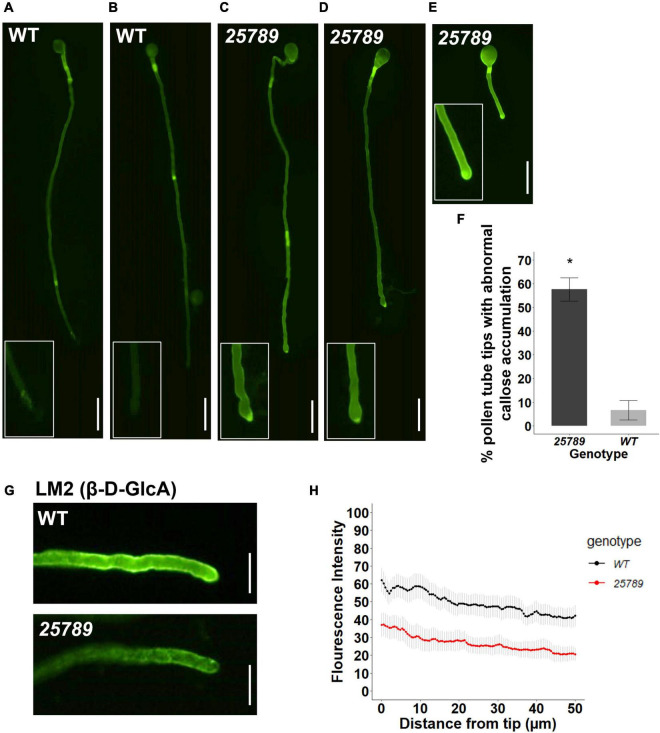
*galt25789* mutant pollen tubes displayed high levels of callose distribution signal at the mutant pollen tube tip and low intensity of LM2 antibody signal during *in vitro* pollen tube growth. **(A,B)** Cytochemical staining of β-glucan (callose) with aniline blue shows WT pollen tube with callose signal in the shaft but lacks staining in the pollen tube tip (enlarged in inset). **(C–E)** displays representative images of abnormal *galt25789* mutant pollen tubes with a higher callose signal at the pollen tube tip than the shaft (enlarged in inset). Quantification of abnormal callose deposition of pollen tube tips is shown in **(F)**. Values are expressed as percentage. Error bars denote mean ± SE, asterisks indicate statistically significant, *p* < 0.005 (*n* ≥ 45 pollen tubes for each genotype). **(G)** Immunofluorescence labeling of cell wall epitopes probed with anti-AGP (β-D-GlcA) MAbs LM2, displays strong staining of pollen tube tip and the surface of pollen tubes in WT. LM2 epitope signal is weaker in *galt25789* mutant pollen tube tip and shaft. **(H)** Quantification of signal intensity from the pollen tube tip to 50 μm down the shaft. Graph represents the mean ± SE of the fluorescent intensities for each genotype (*n* ≥ 25 for each genotype). Scale bars = 50 μm in **(A–E)**, 20 μm in panel **(G)**.

Given that growing pollen tube tip participates in intensive crosstalk with the receptive synergid of ovules, we investigated the cell wall characteristics of pollen tubes. Aniline blue staining showed an atypical abundance of callose labeling at the pollen tube apical and sub-apical regions in *galt25789* quintuple mutants. Approximately 57% of the *galt25789* pollen tubes growing *in vitro* presented callose deposition in the pollen tube tip region while no callose staining was seen in WT pollen tube tips (Figures 8A–F). We observed a strong reduction in LM2 immunolabeling (an AGP-specific mAb that detects β-D-Glc*p*A) at the pollen tube tip in *galt25789* mutants relative to WT (Figure 8G). Quantification of average signal intensity from the pollen tube tip to 50 μm down the shank shows a weaker signal in the apical and sub-apical regions in *galt25789* quintuple mutants when compared to the WT. While JIM7 and JIM5 labeling of *galt25789* mutants were comparable with WT (Supplementary Figure 7).

### Upregulated Expression of Pollen-Specific Glycosylated Arabinogalactan-Proteins in *galt25789* Inflorescences

**FIGURE 9:**
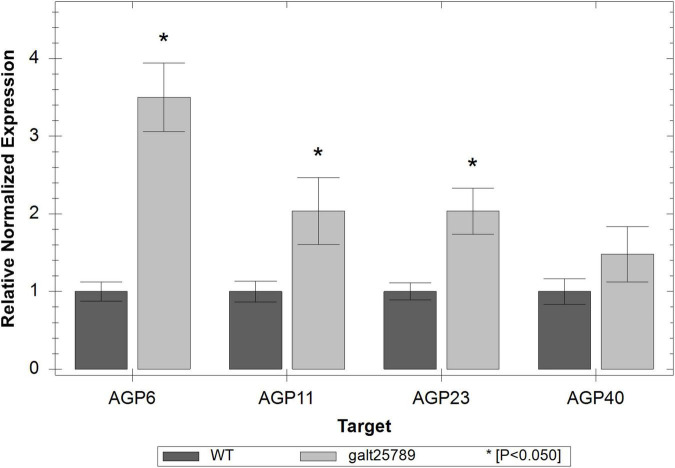
Real time RT-PCR showing relative expression of pollen-specific AGP genes (*AGP6, AGP11, AGP23*, and *AGP40*) in *galt25789* mutants compared to their expression in WT inflorescences. The level of the transcripts was normalized according to the reference genes *ACT8* and *RUB1*. Each bar represents an average of the three technical replicas; the * represents a significant result for *p* < 0.05.

The expression levels of AGP transcripts specific to the male tissues (AGP6, AGP11, AGP23, and AGP40; Coimbra et al., 2009; Nguema-Ona et al., 2012; Costa et al., 2013; da Costa et al., 2013; Pereira et al., 2014) were monitored by Real Time RT-PCR in inflorescences from WT and *galt25789* mutant plants. Real Time RT-PCR analysis showed that these pollen-specific AGPs were significantly up-regulated in the quintuple mutant (Figure 9).

## Discussion

During an extensive phenotypic and biochemical characterization, we previously have shown the *galt25789* quintuple mutant exhibited significant aberrations in seed-set, pollen germination, pollen tube length, and pollen viability with discernible exine defects (Kaur et al., 2021). Despite the large female gametophytic/embryo developmental defects contributing to the reduced seed-set (unpublished data), defects in the male gametogenesis warrant investigation. So far, numerous studies showed how AGPs expressed in spatiotemporal patterns have been implicated in cell fate determination at specific male gametophytic developmental stages (Nguema-Ona et al., 2012; Rafińska et al., 2021). Several AGPs, including AGP23, AGP6, AGP11, and AGP40, is specifically expressed in the male tissues, namely, pollen grains and pollen tubes: (Levitin et al., 2008; Coimbra et al., 2009; Costa et al., 2013; Pereira et al., 2014; Lopes et al., 2019). AGP6 and AGP11 are classical AGPs with functional redundancy and with essential roles in pollen grain development, pollen tube growth, and stamen function (Levitin et al., 2008; Coimbra et al., 2009). A microarray assay together with yeast-2-hybrid experiments showed the involvement of these two AGPs with several members of the PT endosome machinery (Costa et al., 2013). AGP40 is an AG peptide present only in pollen grains and pollen tubes (Nguema-Ona et al., 2012) that has a high similarity to AGP6 and AGP11. The triple mutant *agp6agp11agp40* showed a significant reduction in seed production and early germination of pollen tubes inside the anthers (Costa et al., 2011; Nguema-Ona et al., 2012). Likewise, the viability of the *galt25789* mutant microspores was compromised as revealed by SEM, Alexander staining, and DAPI staining. It is therefore important to tease apart how the presence and/or alterations in type-II AG polysaccharides are critical for AGP function and subsequently affect male fertility in Arabidopsis.

We speculated that *galt25789* mutants produce under-glycosylated AGPs due to the reduction of their sugar additions and likely impair the molecular interactions, leading to the described male function defects. Earlier we reported reduced AGP precipitations by β-Yariv reagent in flower and silique tissues of *galt25789* mutants compared to WT (Kaur et al., 2021). Interestingly, four AGPs (AGP23, AGP6, AGP11, and AGP40) are up-regulated in *galt25789* mutant inflorescences as detected by Real Time RT-PCR experiments. This technique detects mRNA transcripts and not functional proteins, so for this reason we speculate that the *galt25789* mutant might be enhancing the transcription of these AGP genes to compensate for the fact that the AGPs in this mutant are under glycosylated and not normally functioning.

We demonstrated that *galt25789* mutants are critical for the reticulate pollen architecture. In the *galt25789* mutant, the anther area was reduced and many pollen grains were clumped together. An interesting study by Jia et al. (2015) indicated that the expression of four genes encoding AGPs, AGP6, AGP11, AGP23, and AGP40 was regulated by the transcriptional activator, TEK (transposable element silencing *via* AT-hook) in nexine layer formation of the pollen wall. It has been shown previously that the disruption of an anther specific β-(1,3)-galactosyltransferase (KNS4/UPEX1) for AGPs and/or the pectic glycan biosynthesis, demonstrated reduced fertility attributed to anomalous exine design of sterile microspores (Li W.L. et al., 2017; Suzuki et al., 2017). The characteristic exine phenotype of *upex1* and *upex2* mutant microspores was manifested in smaller and shallower baculae with overdeveloped tecta (Dobritsa et al., 2011; Li W.L. et al., 2017; Suzuki et al., 2017). More recently, double, and triple mutants of three glucuronosyl-transferase genes *GLCAT14A*, *GLCAT14B*, and *GLCAT14C* acting on type-II AGPs display disfigured exine marked by wider lacuna with sparse baculae (Ajayi et al., 2021). In contrast, the aberrant *galt25789* pollen exine reticulate patterns were distinct to an extent where the denser baculae carved out smaller lacunae and congested tectum. Besides these sculpting differences, the *galt25789* mutant exine features were analogous to the uneven exine phenotype of *kns4* and *glcat14a/b/c* mutants with regard to the absence of reticulate structure in extreme cases and naked sticky pollen scraps left after crumpling of the uneven exine structure. An interesting study on apyrases AtAPY6/7, which are speculated to modulate glycosylation of glycoproteins through the adjustment of NDP concentrations in the ER or Golgi, revealed severely deformed pollen grains with abnormal exine reticulate patterns in double mutants (Yang et al., 2013). These overlapping and unique exine architectural observations reinforce the concept that the Hyp-GALTs (that initiate the synthesis of the Hyp-*O*-glycans) along with KNS4/UPEX and GLCAT14A, B, C (that further elongate and decorate the Hyp-*O*-glycans) presumably maintain the pollen cell wall architecture.

The intricately designed pollen exine matrix enveloping the microspore is supported underneath by a much simpler intine layer, mainly consisting of cellulose, hemicellulose, pectin, and structural proteins; the intine layer development is predominantly completed by the end of the binucleate stage (Quilichini et al., 2015; Shi et al., 2015; Ma et al., 2021). The intine structure is more complex at the site of apertures and furrows where the pollen tube emerge (Wang and Dobritsa, 2018); hence intine layer defects could possibly affect the germination process. And this is consistent with our observations on pollen grains exhibiting slightly flatter aperture ridges and low pollen germination in *galt25789* mutants (Kaur et al., 2021). Two fasciclin-like AGPs, FLA3 (Li et al., 2010) and FLA14 (Miao et al., 2021) were shown to be involved in pollen development, resulting in altered intine thickness and pollen collapsing events. In other studies, with *Brassica campestris*, antisense RNA mutants of a pollen-specific AGP encoding gene, BcMF8 (*B. campestris* male fertility 8; Huang et al., 2008; Lin et al., 2014) and BcMF18 mutants (Lin et al., 2018) produced deformed pollen grains with under built intine, cytoplasm, and nuclei. Our TEM results also showed a thin, uneven, and disrupted pollen intine layer in the *galt25789* mutants. And in severe cases, pollen collapsing events occurred from extensive degeneration and shrinkage of cytoplasmic contents in the quintuple mutants. The intine layer is indispensable for pollen architecture as evidenced by work on rice Glycosyltransferase 1 (OsGT1; Moon et al., 2013) and rice pollen-specific arabinokinase-like protein collapsed abnormal pollen 1 (CAP1) mutants (Ueda et al., 2013) which displayed distorted intine. Also, a *glcat14a/b/c* mutant study reported the significance of AG glucuronidation of type-II AGs for Arabidopsis intine development (Ajayi et al., 2021). All these observations together with our results indicate that the shriveled pollen with poorly developed intine might have resulted from disordered pollen cell wall polysaccharides assembly due to loss of classic type-II AGs, which define their systemic and signaling functions for AGPs.

While inspecting the *galt25789* pollen grain in anther section, we noticed more lytic vacuoles in the pollen cytoplasm of collapsed pollen grains, indicative of abnormal metabolic activity that might have resulted in pollen abortion. Generally, dynamic vacuolar fusion and fission processes are known to occur during pollen development (Yamamoto et al., 2003). Typical WT pollen contains a large vegetative vacuole formed in a uninucleate microspore that disappears in the bicellular stage. Big lytic vacuoles accompanied by degraded cytoplasm are responsible for the loss of the ability of pollen germination after anthesis, eventually leading to pollen grain autolysis (Yamamoto et al., 2003; Zhang et al., 2018). Coimbra et al. (2009) reported similar microscopic observations for *agp6 agp11* aborted pollen grains, which displayed condensed cytoplasm, membrane blebbing, and the presence of lytic vacuoles. Moreover, vacuolar disorganization appeared as excess and/or enlarged vacuoles in the swollen tapetal layer at different stages of *galt25789* anther development compared to WT from flower stage 8–12. Given that the tapetal differentiation and timely degeneration of tapetal cells through programmed cell death (PCD) is a prerequisite for the supply of nutrients to produce functional microspores, particularly with respect to the formation of pollen exine (Zhou et al., 2012; Zhang et al., 2014; Cheng et al., 2020), we suggested a functional role for type-II AGs in both tapetal layer and pollen grain differentiation.

Previously reported *ams* mutants (encoding a defective bHLH transcription factor) presented similar phenotypes with abnormal vacuolization and hypertrophy of the tapetal cells, which caused premature degradation of microspores. In fact, AMS is a master switch for a regulatory cascade involving the expression of 23 diverse genes for pollen wall development; one of those targets is the TEK promoter, which is further known to regulate several AGPs involved in the biosynthesis of the nexine and AtMYB103/MYB80 for sexine formation (Lou et al., 2014, 2018; Ferguson et al., 2017; Xiong et al., 2020). As Arabidopsis tapetum development and function are known to be regulated by DYSFUNCTIONAL TAPETUM1 (DYT1), a putative bHLH transcription factor (Zhang et al., 2006), and Arabidopsis DYSFUNCTIONAL TAPETUM 1, (TDF1) acts upstream of AMS (Zhu et al., 2008); thus, we further propose that the established DYT1-TDF1-AMS-TEK/MYB103/MYB80-AGPs genetic pathway regulates pollen wall development (Shi et al., 2015; Ma et al., 2021) through interactive molecular surfaces provided by the type-II Hyp-*O*-glycans.

Our results from AGP immunolabelling of anther sections in this study corroborate our previous results for *galt25789* mutant AGPs to be underglycosylated (as indicated by severe reduction in β-Yariv precipitable AGPs of mutants; Kaur et al., 2021). We detected slight differences in AGP epitopes recognized by the JIM13 mAb, which were less intense around the wall in PMC, tapetal cell layer and mature microspores of *galt25789* mutants than WT. Interestingly, JIM8 immunolabelling in aborted pollen grains of *galt25789* mutants was quite intense, possibly reflecting the abnormal deposition of the AGP glycan moieties during male gametogenesis. Such discrepancies in microsporocytes immunolabeled with AGP mAbs (JIM8 and JIM13) were seen before by others (Coimbra et al., 2007; Suzuki et al., 2017; Ajayi et al., 2021). Despite the diagnostic feature of mAbs providing specificity for tissue-specific carbohydrate epitopes on multiple AGPs with differentially glycosylated protein cores, these experiments depend on the characterization of a set of AGPs with epitopes in that tissue. Hence, low signals indicate under-glycosylation of various AGPs. A strong reduction in LM2 labeling for all developmental stages was displayed in the *galt25789* mutants, indicating that the relative abundance of methyl-ß-D-GlcA or GlcA AGP epitopes decreased considerably in the mutant in comparison to the WT. Similar pollen abortion phenotypes and weaker LM2 immunolabeling patterns in anther sections were observed previously in the *glcat14a/b/c* mutants (Ajayi et al., 2021). In this work, we detected only slight differences in the less-methylesterified HG (JIM5) in the walls of anther and pollen in *galt25789* anthers while highly methylesterified HG pectin (JIM7) appeared to be deposited in a normal manner in *galt25789* mutant microspores like the WT with relatively uniform signals. Similar observations for a weaker JIM5 signal were made by Suzuki et al. (2017). Likewise, Li W.L. et al. (2017) have shown that the alterations in wall polysaccharides like xylan and AGPs affect the microspore primexine. Given the fact that pectins are initially secreted in the methylesterified form and then demethylesterified in the wall by pectin methyl esterases (PMEs; Zhang and Staehelin, 1992), our results indicate that the altered dynamics of AGP synthesis and glycosylation patterns affects the other cell wall components like pectin and as such warrants further investigation.

GlcA residue is considered to play a mechanistic role in the prevailing AGP-Ca^2+^ capacitor model by generating Ca^2+^ oscillations in a pH-dependent manner (Lamport and Várnai, 2013; Lamport et al., 2014). Our observation with LM2 also suggests that a reduction in fully glycosylated AGPs in the pollen tubes might be responsible for the change in the cell wall integrity of pollen tube growth. Furthermore, callose is also a small portion of the cell wall polymers in pollen tubes, and callose deposition can be enhanced by a variety of stimuli, including cytosolic calcium levels (Bhuja et al., 2004). Such alteration in the dynamics of the pollen tube cell wall is likely associated with a male–female cross talk for the fertilization process.

In summary, the work here established the involvement of AGP Hyp-*O*-glycans in the male reproductive organ development, specifically exine and intine of pollen grains. We propose that the under-glycosylated AGPs and overall reduced amount of glycosylated AGPs in the reproductive organs not only perturb the structural aspects of the male sporophytic cell walls (endothecium) but also the nutrition sources (tapetum) for male gametophytic phases. Additionally, the lytic vacuoles inside mature pollen grains increase in number, presumably for degradation of under-glycosylated AGPs. Moreover, under-glycosylated AGPs in pollen tube cell walls and callose deposition at the apical region suggested that AGPs might be involved in providing signaling cues for male-female interactions essential for successful reproduction.

## Data Availability Statement

The raw data supporting the conclusions of this article will be made available by the authors, without undue reservation.

## Author Contributions

DK designed the research, analyzed the data, wrote the initial draft of the manuscript, and conducted all microscopic experiments (SEM, TEM, stereomicroscopy, epifluorescence, and confocal microscopy). DM conducted RT-qPCR and wrote for this experiment of the manuscript. SC and AS conceived the study, helped to analyze and interpret the data, and were involved in reviewing the subsequent drafts of the manuscript. All authors have read and approved the manuscript.

## Conflict of Interest

The authors declare that the research was conducted in the absence of any commercial or financial relationships that could be construed as a potential conflict of interest.

## Publisher’s Note

All claims expressed in this article are solely those of the authors and do not necessarily represent those of their affiliated organizations, or those of the publisher, the editors and the reviewers. Any product that may be evaluated in this article, or claim that may be made by its manufacturer, is not guaranteed or endorsed by the publisher.
