# Supplementary material for: Hydroxyproline-O-Galactosyltransferases Synthesizing Type II Arabinogalactans Are Essential for Male Gametophytic Development in Arabidopsis
Source: Front Plant Sci. 2022 Jun 14;13:935413. doi: 10.3389/fpls.2022.935413 (PMC9237623; doi:10.3389/fpls.2022.935413)
Supplement: Supplementary file 1 [file Presentation_1.pptx]

## Slide 1
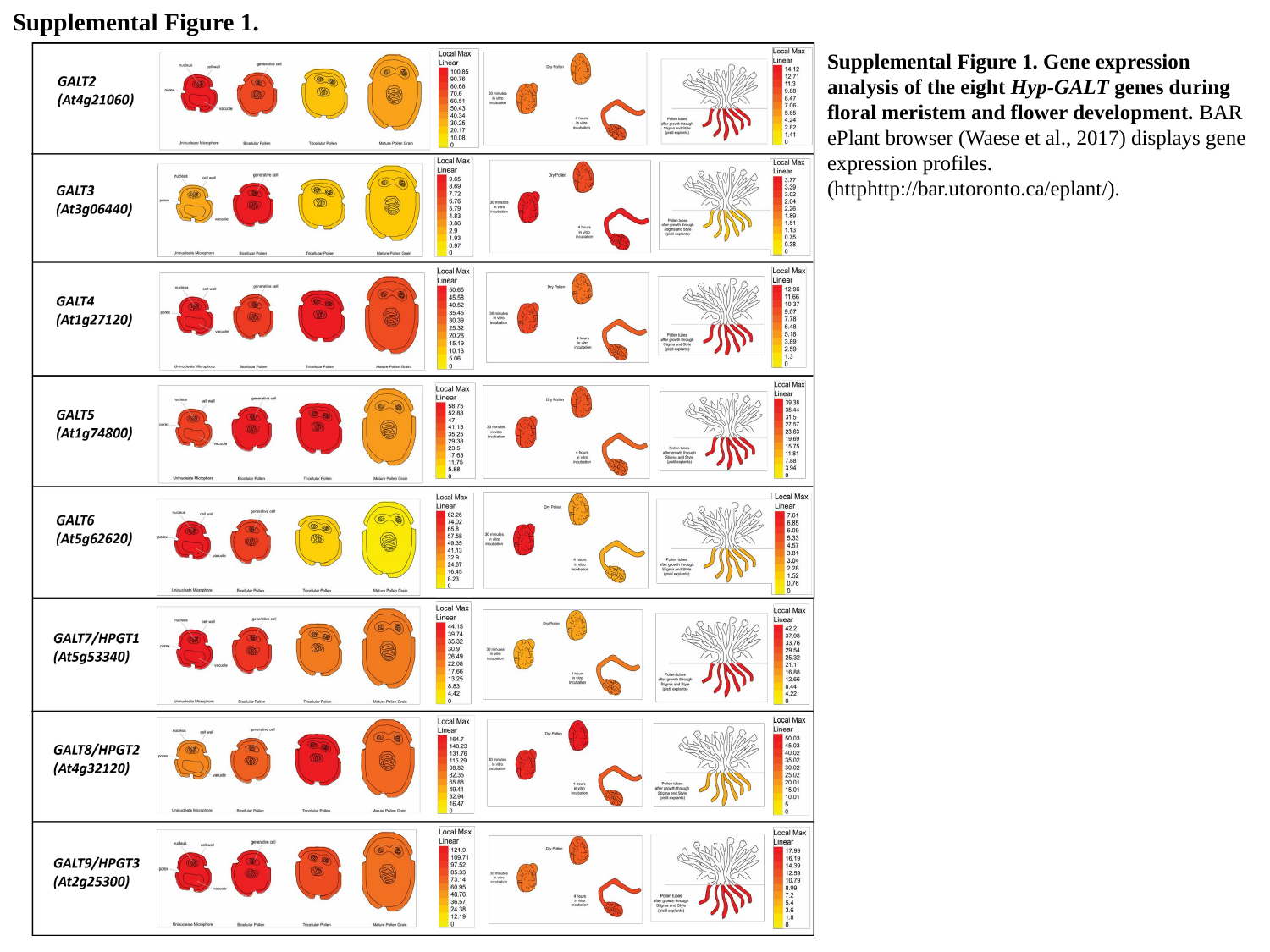

Supplemental Figure 1.
Supplemental Figure 1. Gene expression analysis of the eight Hyp-GALT genes during floral meristem and flower development. BAR ePlant browser (Waese et al., 2017) displays gene expression profiles. (httphttp://bar.utoronto.ca/eplant/).

## Slide 2
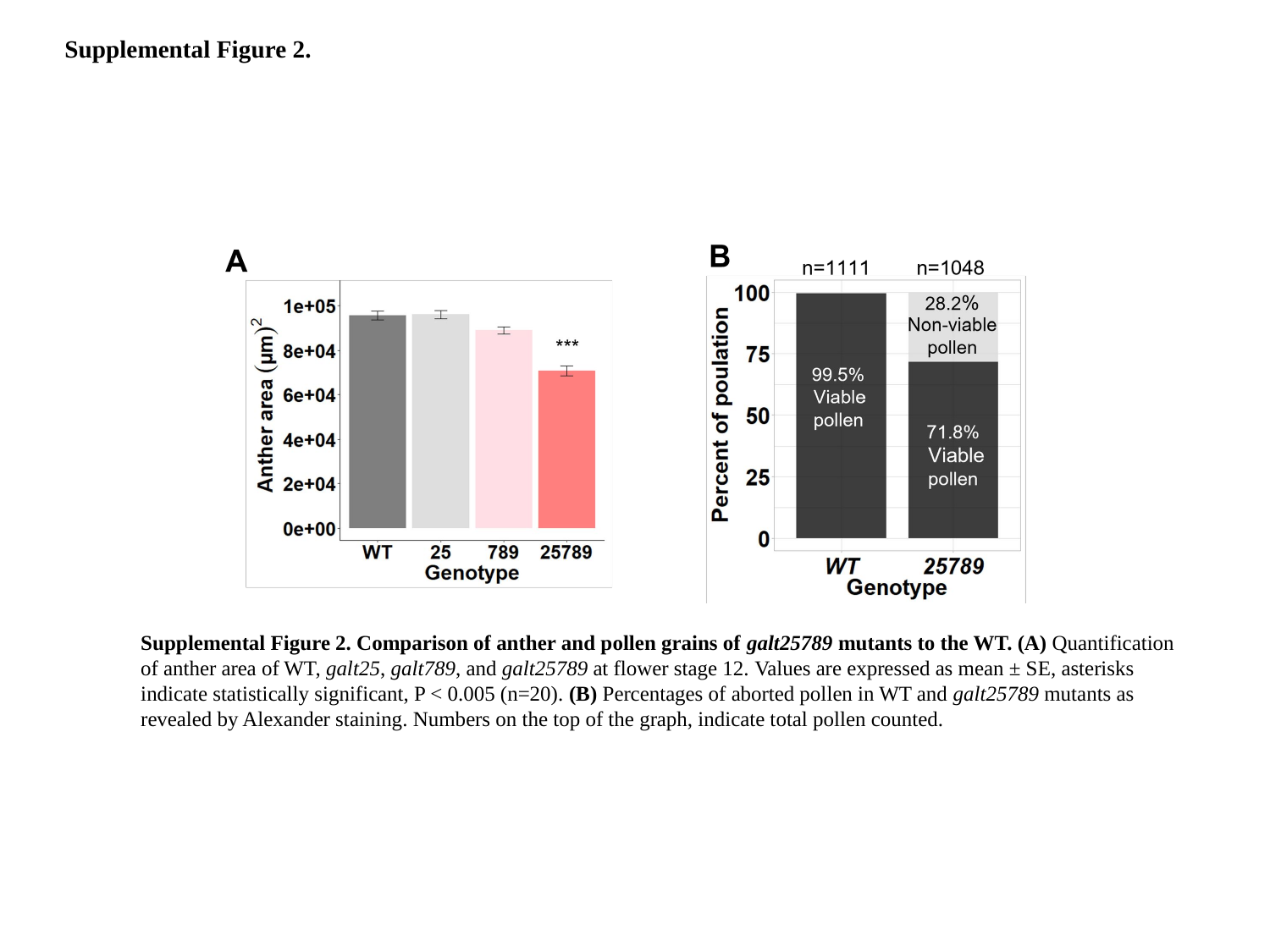

Supplemental Figure 2.
Supplemental Figure 2. Comparison of anther and pollen grains of galt25789 mutants to the WT. (A) Quantification of anther area of WT, galt25, galt789, and galt25789 at flower stage 12. Values are expressed as mean ± SE, asterisks indicate statistically significant, P < 0.005 (n=20). (B) Percentages of aborted pollen in WT and galt25789 mutants as revealed by Alexander staining. Numbers on the top of the graph, indicate total pollen counted.

## Slide 3
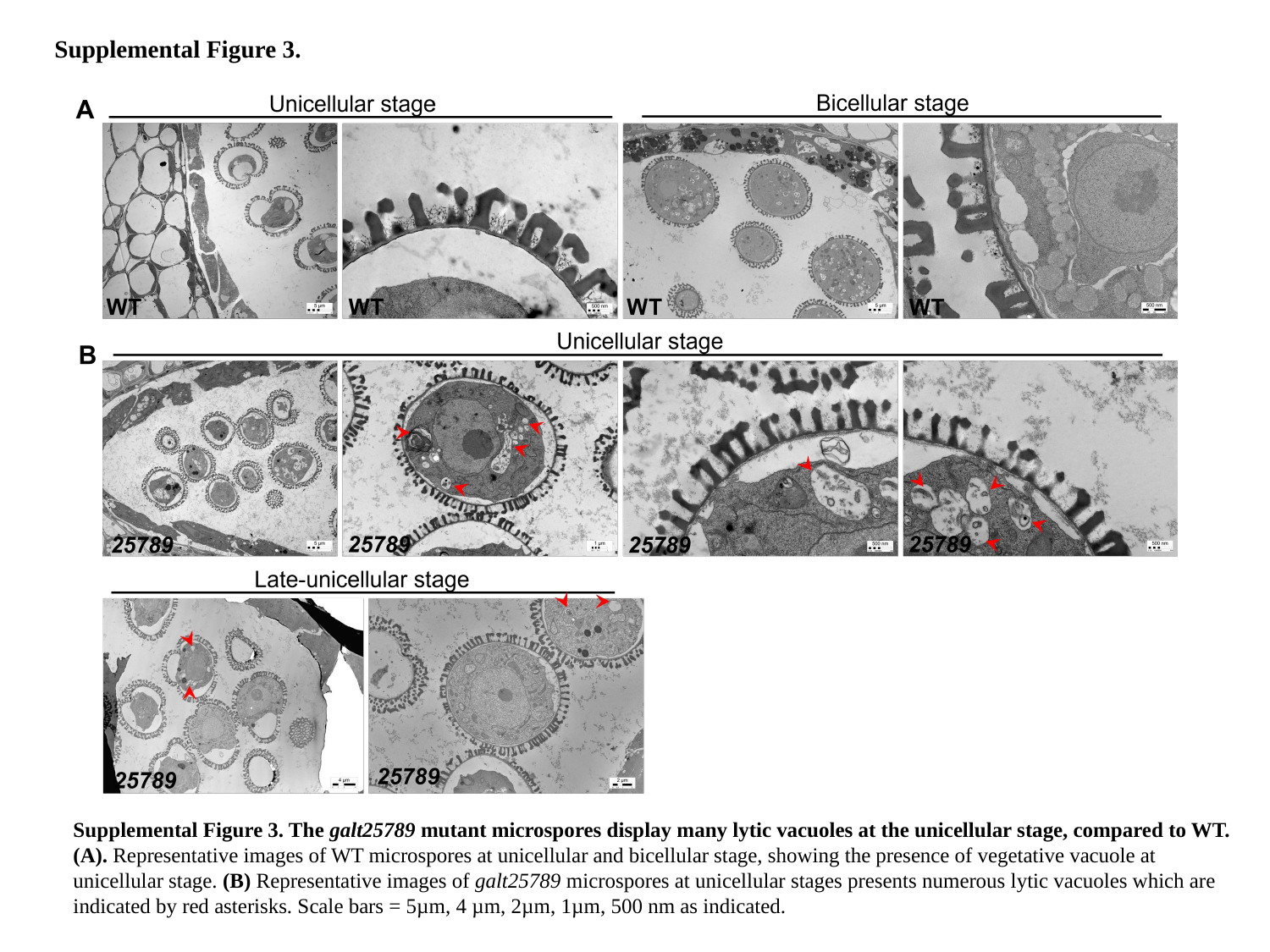

Supplemental Figure 3.
Supplemental Figure 3. The galt25789 mutant microspores display many lytic vacuoles at the unicellular stage, compared to WT. (A). Representative images of WT microspores at unicellular and bicellular stage, showing the presence of vegetative vacuole at unicellular stage. (B) Representative images of galt25789 microspores at unicellular stages presents numerous lytic vacuoles which are indicated by red asterisks. Scale bars = 5µm, 4 µm, 2µm, 1µm, 500 nm as indicated.

## Slide 4
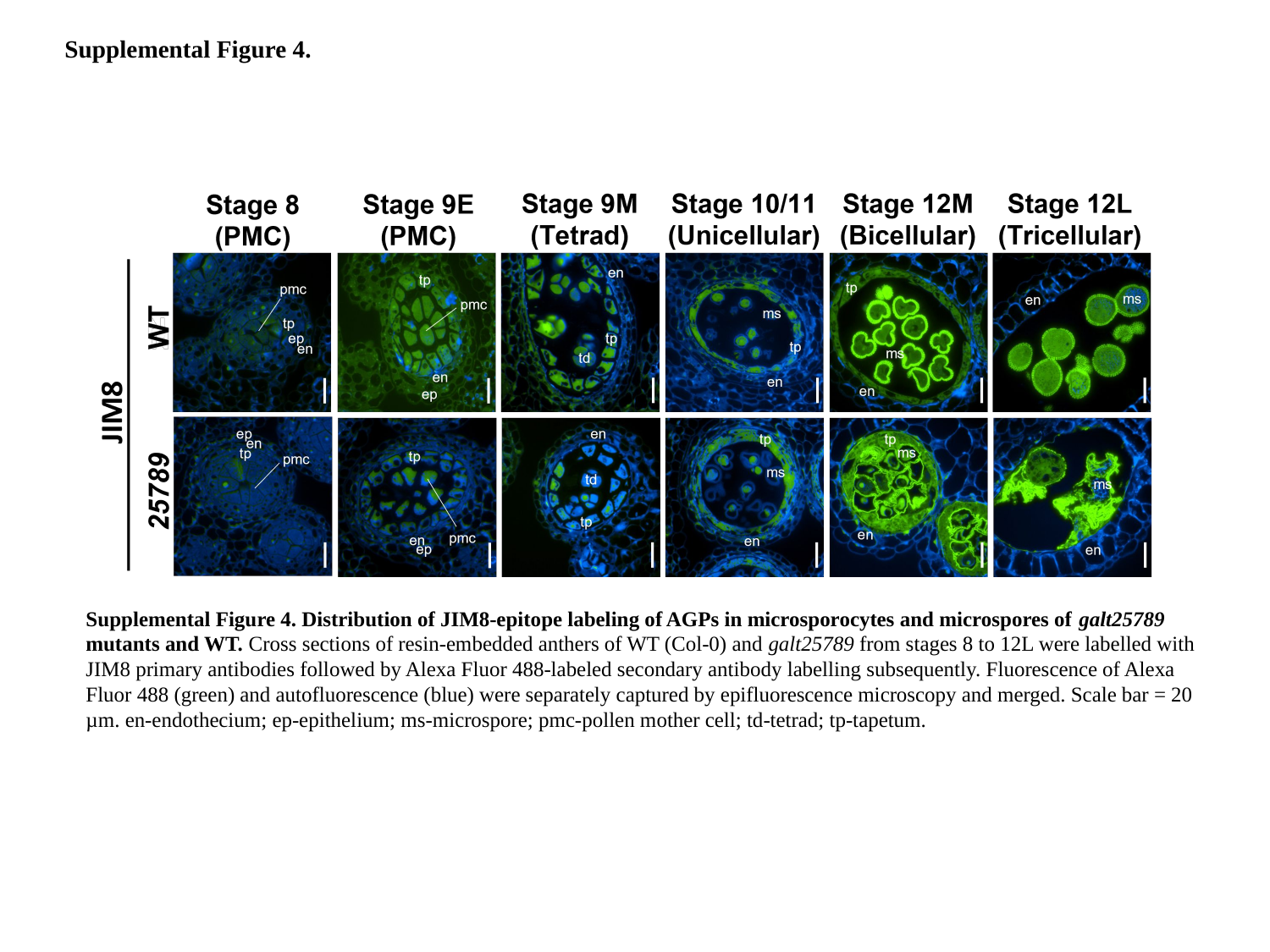

Supplemental Figure 4.
Supplemental Figure 4. Distribution of JIM8-epitope labeling of AGPs in microsporocytes and microspores of galt25789 mutants and WT. Cross sections of resin-embedded anthers of WT (Col-0) and galt25789 from stages 8 to 12L were labelled with JIM8 primary antibodies followed by Alexa Fluor 488-labeled secondary antibody labelling subsequently. Fluorescence of Alexa Fluor 488 (green) and autofluorescence (blue) were separately captured by epifluorescence microscopy and merged. Scale bar = 20 µm. en-endothecium; ep-epithelium; ms-microspore; pmc-pollen mother cell; td-tetrad; tp-tapetum.

## Slide 5
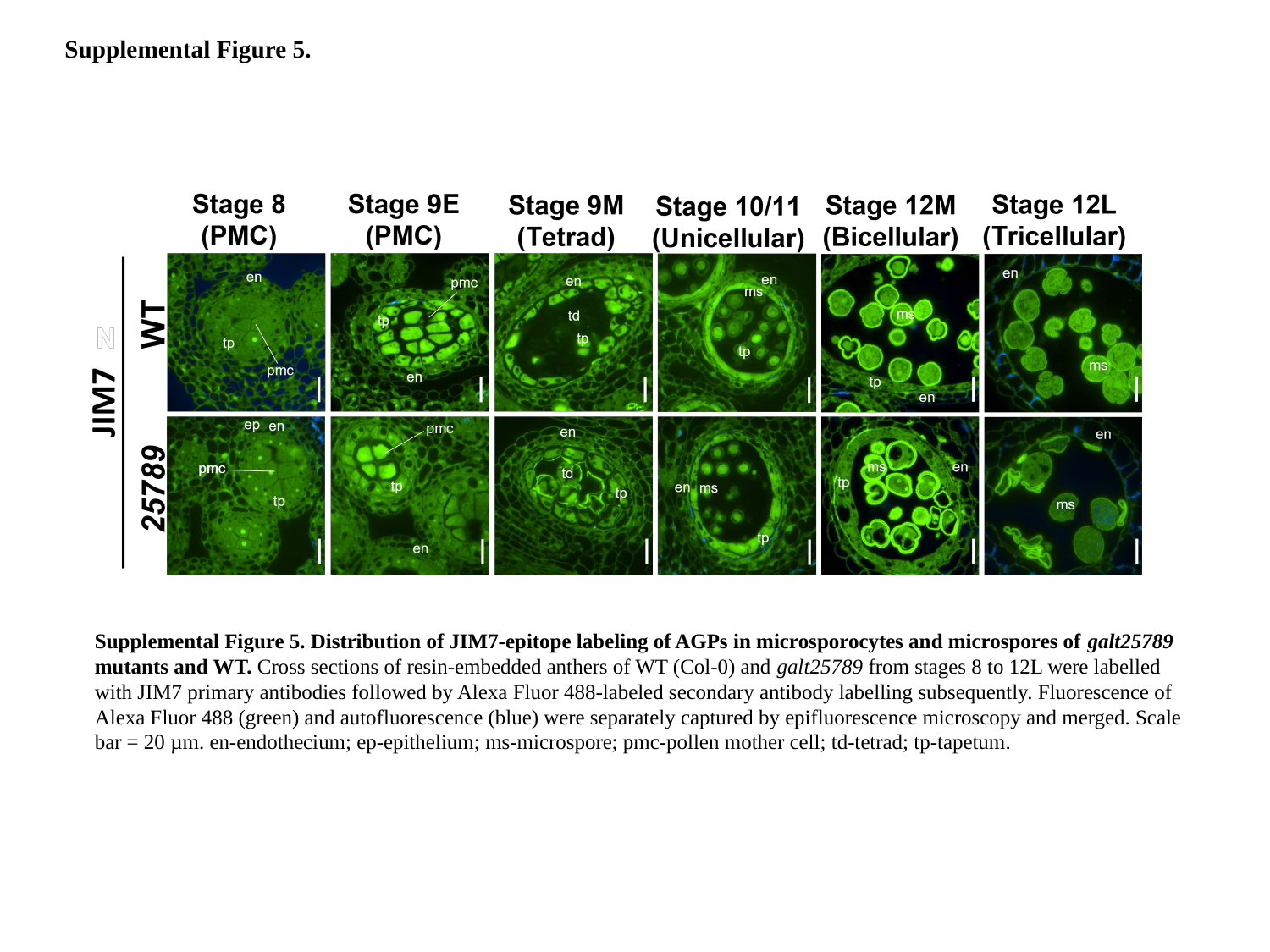

Supplemental Figure 5.
Supplemental Figure 5. Distribution of JIM7-epitope labeling of AGPs in microsporocytes and microspores of galt25789 mutants and WT. Cross sections of resin-embedded anthers of WT (Col-0) and galt25789 from stages 8 to 12L were labelled with JIM7 primary antibodies followed by Alexa Fluor 488-labeled secondary antibody labelling subsequently. Fluorescence of Alexa Fluor 488 (green) and autofluorescence (blue) were separately captured by epifluorescence microscopy and merged. Scale bar = 20 µm. en-endothecium; ep-epithelium; ms-microspore; pmc-pollen mother cell; td-tetrad; tp-tapetum.

## Slide 6
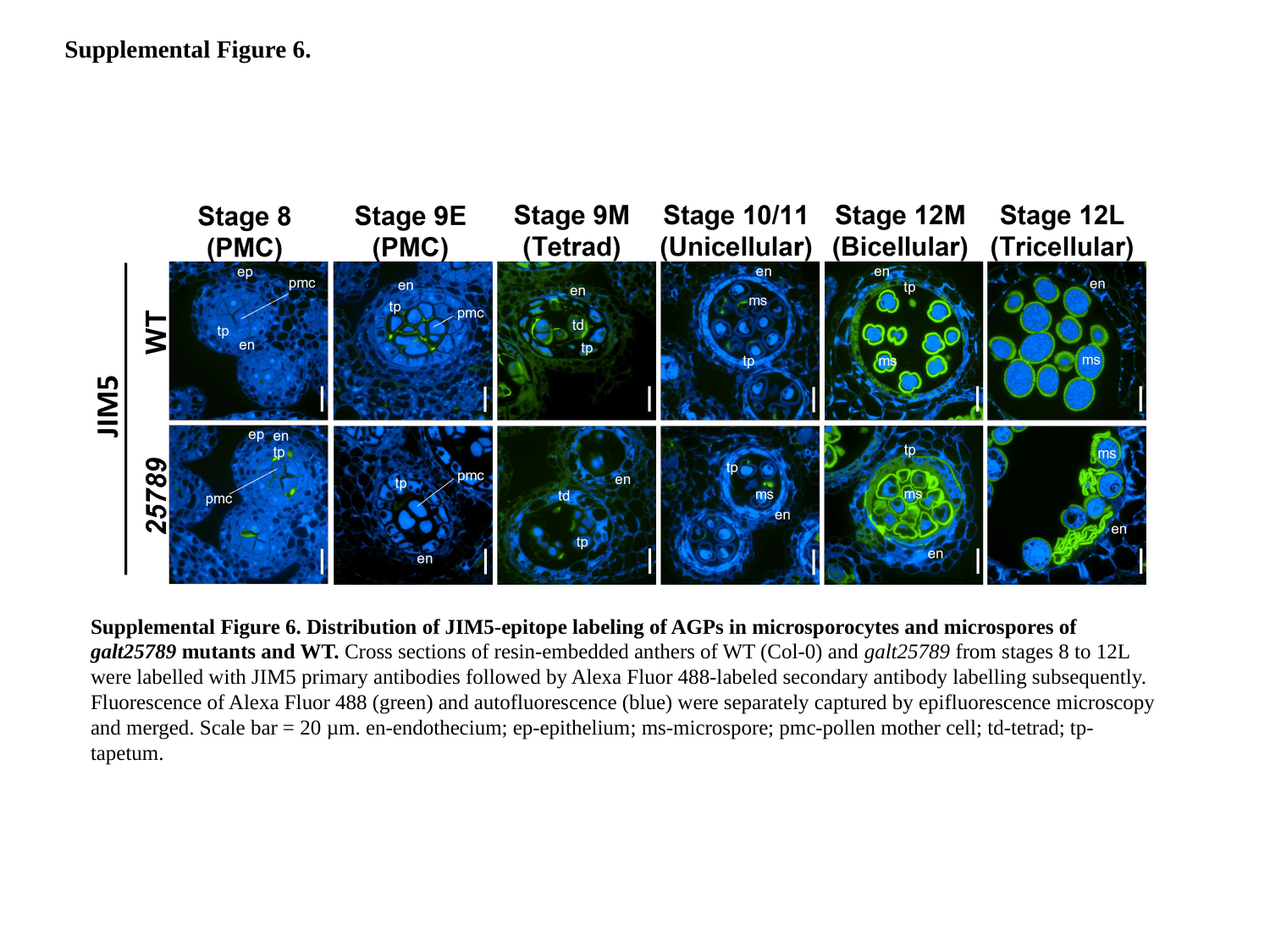

Supplemental Figure 6.
Supplemental Figure 6. Distribution of JIM5-epitope labeling of AGPs in microsporocytes and microspores of galt25789 mutants and WT. Cross sections of resin-embedded anthers of WT (Col-0) and galt25789 from stages 8 to 12L were labelled with JIM5 primary antibodies followed by Alexa Fluor 488-labeled secondary antibody labelling subsequently. Fluorescence of Alexa Fluor 488 (green) and autofluorescence (blue) were separately captured by epifluorescence microscopy and merged. Scale bar = 20 µm. en-endothecium; ep-epithelium; ms-microspore; pmc-pollen mother cell; td-tetrad; tp-tapetum.

## Slide 7
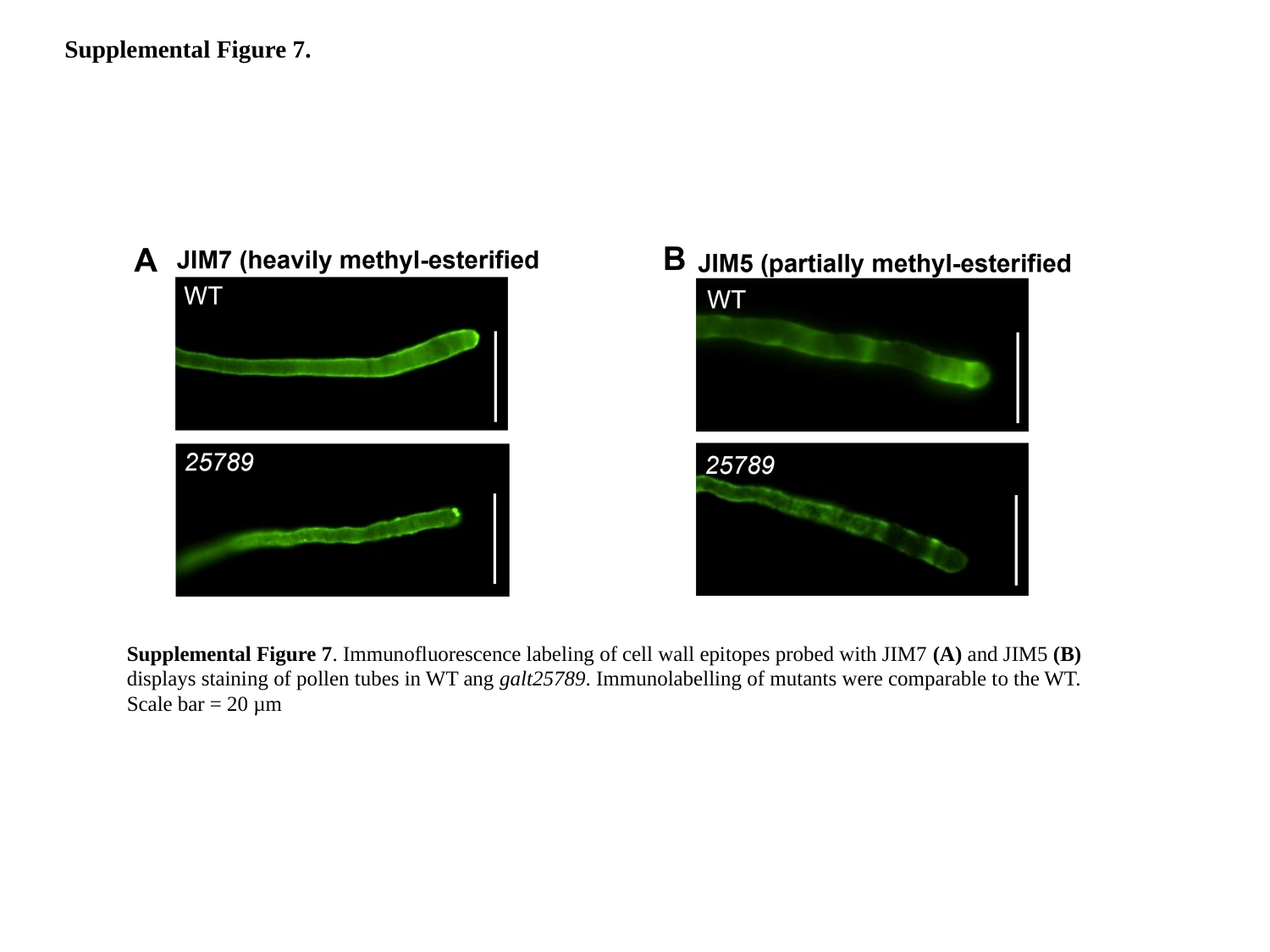

Supplemental Figure 7.
Supplemental Figure 7. Immunofluorescence labeling of cell wall epitopes probed with JIM7 (A) and JIM5 (B) displays staining of pollen tubes in WT ang galt25789. Immunolabelling of mutants were comparable to the WT. Scale bar = 20 µm

## Slide 8
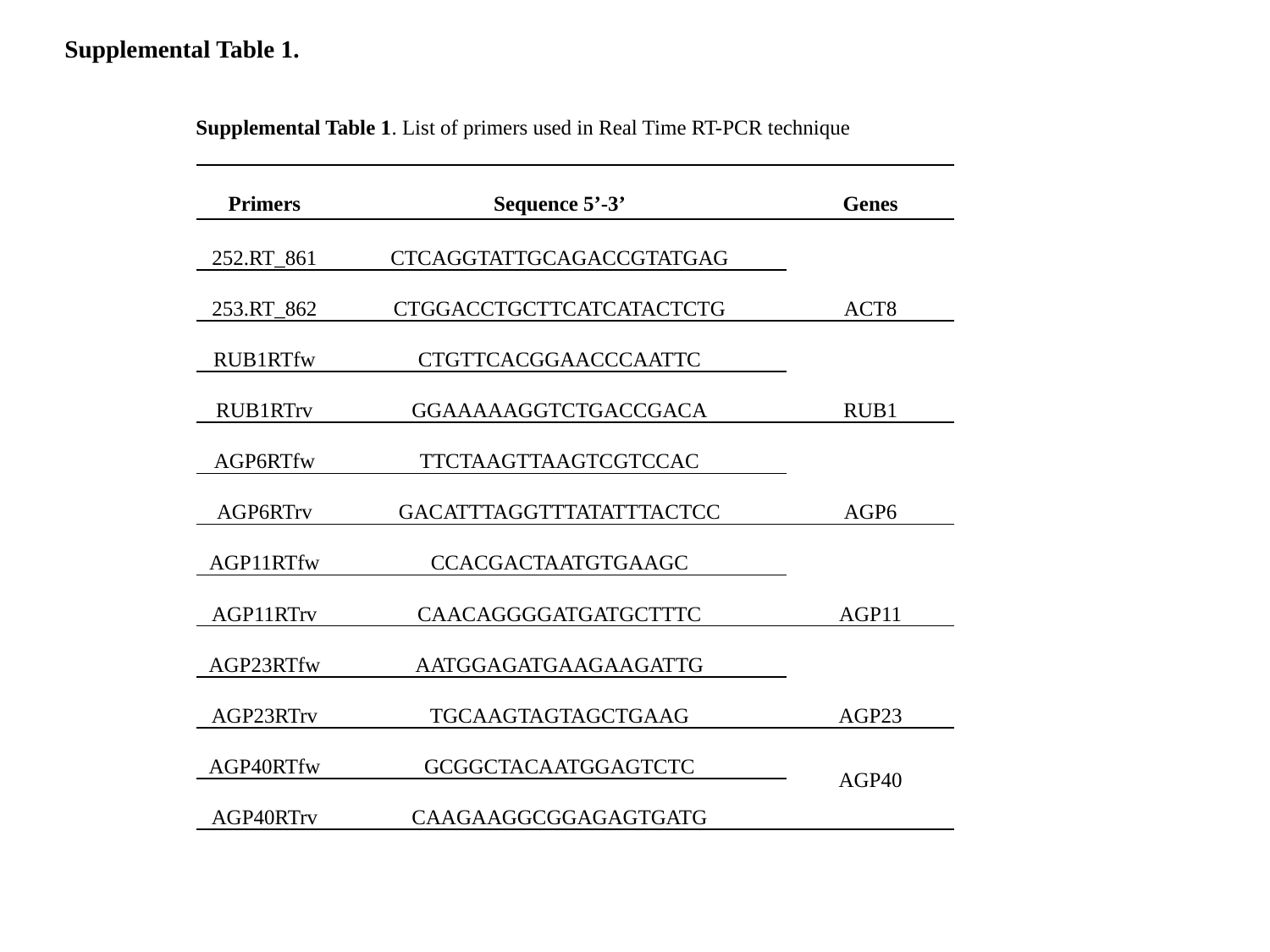

Supplemental Table 1.
Supplemental Table 1. List of primers used in Real Time RT-PCR technique
| Primers | Sequence 5’-3’ | Genes |
| --- | --- | --- |
| 252.RT\_861 | CTCAGGTATTGCAGACCGTATGAG | ACT8 |
| 253.RT\_862 | CTGGACCTGCTTCATCATACTCTG | |
| RUB1RTfw | CTGTTCACGGAACCCAATTC | RUB1 |
| RUB1RTrv | GGAAAAAGGTCTGACCGACA | |
| AGP6RTfw | TTCTAAGTTAAGTCGTCCAC | AGP6 |
| AGP6RTrv | GACATTTAGGTTTATATTTACTCC | |
| AGP11RTfw | CCACGACTAATGTGAAGC | AGP11 |
| AGP11RTrv | CAACAGGGGATGATGCTTTC | |
| AGP23RTfw | AATGGAGATGAAGAAGATTG | AGP23 |
| AGP23RTrv | TGCAAGTAGTAGCTGAAG | |
| AGP40RTfw | GCGGCTACAATGGAGTCTC | AGP40 |
| AGP40RTrv | CAAGAAGGCGGAGAGTGATG | |
